# HTLV-1 bZIP factor supports proliferation of adult T cell leukemia cells through suppression of C/EBPα signaling

**DOI:** 10.1186/1742-4690-10-159

**Published:** 2013-12-21

**Authors:** Tiejun Zhao, Aaron Coutts, Lingling Xu, Juntao Yu, Koichi Ohshima, Masao Matsuoka

**Affiliations:** 1 College of Chemistry and Life Sciences, Zhejiang Normal University, 688 Yingbin Road, Jinhua, Zhejiang 321004, China; 2 Laboratory of Virus Control, Institute for Virus Research, Kyoto University, 53 Shogoin Kawahara-cho, Sakyo-ku, Kyoto 606-8507, Japan; 3 Department of Pathology, School of Medicine, Kurume University, 67 Asahimachi, Kurume, Fukuoka 830-0011, Japan; 4 Present address: School of Medicine, The University of Queensland, Herston 4006, Australia

**Keywords:** HTLV-1, HBZ, C/EBPα

## Abstract

**Background:**

Human T-cell leukemia virus type 1 (HTLV-1) is an oncogenic retrovirus etiologically associated with adult T-cell leukemia (ATL). The HTLV-1 bZIP factor (HBZ), which is encoded by minus strand of provirus, is expressed in all ATL cases and supports the proliferation of ATL cells. However, the precise mechanism of growth promoting activity of HBZ is poorly understood.

**Results:**

In this study, we showed that HBZ suppressed C/EBPα signaling activation induced by either Tax or C/EBPα. As mechanisms of HBZ-mediated C/EBPα inhibition, we found that HBZ physically interacted with C/EBPα and diminished its DNA binding capacity. Luciferase and immunoprecipitation assays revealed that HBZ repressed C/EBPα activation in a Smad3-dependent manner. In addition, C/EBPα was overexpressed in HTLV-1 infected cell lines and fresh ATL cases. HBZ was able to induce C/EBPα transcription by enhancing its promoter activity. Finally, HBZ selectively modulated the expression of C/EBPα target genes, leading to the impairment of C/EBPα-mediated cell growth suppression.

**Conclusion:**

HBZ, by suppressing C/EBPα signaling, supports the proliferation of HTLV-1 infected cells, which is thought to be critical for oncogenesis.

## Background

Human T-cell leukemia virus type 1 (HTLV-1) is the causative agent of adult T-cell leukemia (ATL) [1,2]. HTLV-1 encodes several regulatory (*tax* and *rex*) and accessory (*p12*, *p13* and *p30*) genes in the pX region located between the *env* and 3’ long terminal repeat (LTR) [3]. Among the viral genes, Tax is thought to play a central role in the pathogenesis of HTLV-1 [4]. Yet the expression of Tax cannot be detected in ~60% of fresh ATL cases due to epigenetic modifications or deletion of the 5’LTR [5]. In contrast, the *HTLV-1 bZIP factor* (*HBZ*), which is encoded by the minus strand of the HTLV-1 genome, is expressed in all ATL cases and supports the proliferation of HTLV-1 infected cells [6-8]. HTLV-2, a type of retrovirus which is similar with HTLV-1, encodes an antisense protein (APH-2) using the minus strand of its genome. However, APH-2 does not seem to promote cell proliferation [9,10]. HBZ was reported to repress Tax-mediated transactivation of viral transcription from the HTLV-1 5’LTR [11]. Moreover, HBZ dysregulated multiple cellular signalings including the classical pathway of NF-κB, TGF-β, AP-1, and the Wnt pathways, which is likely to contribute to viral persistence and clonal expansion of infected cells [12-15].

The CCAAT/enhancer binding protein (C/EBP) family of proteins represents a critical group of bZIP transcription factors that are key to the regulation of cell proliferation, development, and immune responses [16,17]. Dysregulated C/EBP signaling is intimately associated with tumorigenesis and viral diseases [18]. Furthermore, the ability of C/EBPs to direct cellular fate is thought to depend on the presence of specific collaborating transcription factors, and allows C/EBPs to act as both tumor suppressors and tumor promoters under different conditions [17]. C/EBPα, the founding member of this family, has been demonstrated to be important for differentiation of several cell types [19]. On the other hand, C/EBPα emerged as an important negative regulator of cell proliferation [20]. Thus, most tumors have evolved distinct strategies to attenuate C/EBPα function [17,21]. Known mechanisms of C/EBPα suppression in cancer cells include (1) transcriptional downregulation of *CEBPA* expression; (2) point mutations and deletions in C/EBPα; and (3) inhibition of C/EBPα transcriptional activation through protein-protein interaction. However, normal C/EBPα is overexpressed in B-cell precursor acute lymphoblastic leukemia (BCP-ALL), and inhibits apoptosis by upregulating bcl-2 and FLIP expression [22,23]. It suggested that C/EBPα may exhibit oncogenic as well as tumour suppressor properties in human leukaemogenesis.

In ATL, Tax has been shown to bind to CCAAT binding proteins such as nuclear factor YB subunit (NF-YB) and C/EBPβ [24]. Through its association with NF-YB, Tax activates the major histocompatibility complex class II (MHC-II) promoter [24]. Additionally, C/EBPβ was capable of inhibiting Tax-dependent transactivation of the HTLV-1 LTR, as well as efficiently decreasing Tax synthesis from an infectious HTLV-1 molecular clone [25]. On the other hand, expression of Tax increases binding of C/EBPβ to and activates the IL-1β promoter [26]. Interestingly, previously published microarray data showed that the *CEBPA* gene was overexpressed in adult T-cell leukemia cells [27,28]. It is thus likely that the dysregulated C/EBP signaling pathway may play a role in ATL.

Although regulation of C/EBP signaling by Tax has been reported, little is known about whether other viral proteins affect C/EBP signaling. In the present study, we found that HBZ suppressed C/EBP signaling by interacting with C/EBPα, resulting in the impairment of C/EBPα-mediated cell growth suppression. This might account for why HBZ supports the proliferation of HTLV-1 infected cells.

## Results

### HBZ suppresses C/EBPα signaling

**Figure 1:**
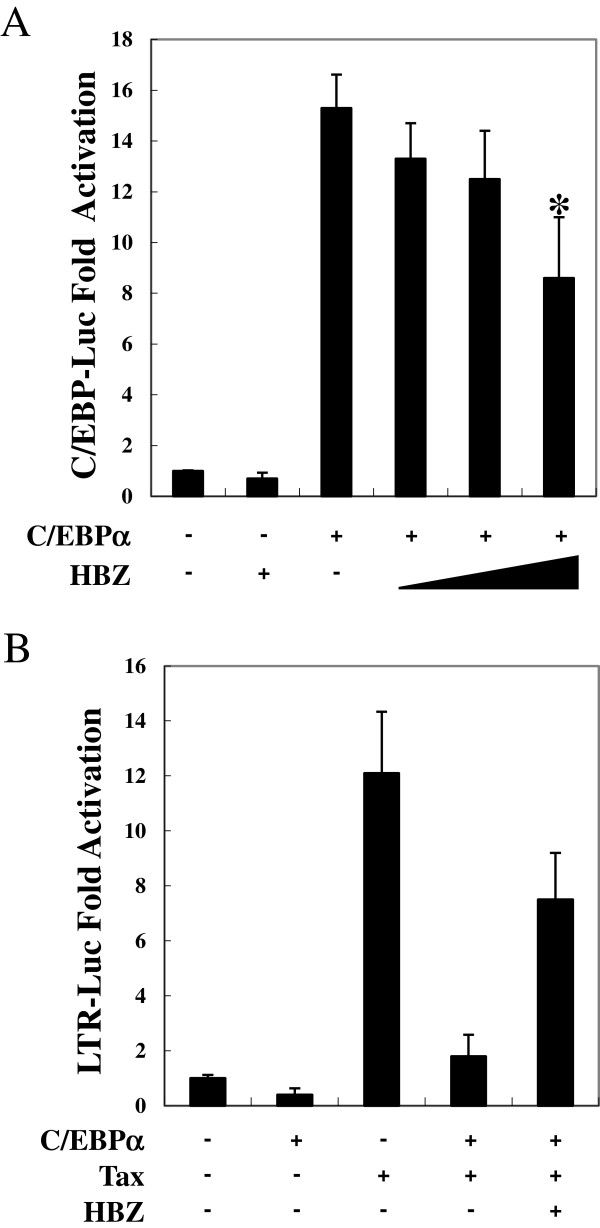
**HBZ suppressed C/EBPα signaling. (A)** HBZ repressed C/EBPα-induced transcriptional activation. Jurkat cells were cotransfected with pC/EBP-Luc (0.5 μg), phRL-TK (10 ng), pME18Sneo-HBZ (0, 0.5, 1, and 2 μg), and pCMV-Tag-C/EBPα (1 μg). After 48 hours, the cells were harvested and analyzed for luciferase activity. **(B)** HBZ impaired the suppressive effect of C/EBPα on HTLV-1 LTR activation. Jurkat cells were cotransfected with pLTR-Luc (0.5 μg), phRL-TK (10 ng), and pME18Sneo-HBZ (2 μg), pCG-Tax (1 μg), together with pCMV-Tag-C/EBPα (1 μg). At 48 hours after transfection, a dual luciferase reporter assay was performed. All the data shown are relative values of firefly luciferase normalized to Renilla luciferase and expressed as mean of a triplicate set of experiments ± SD. *P < 0.05; **P < 0.01.

To investigate the effect of HBZ on the C/EBP signaling pathway, Jurkat cells were cotransfected with expression vectors of C/EBPα and HBZ along with a C/EBP-responsive reporter: C/EBP-Luc. As shown in Figure 1A, C/EBPα enhanced the transcription of luciferase, while HBZ inhibited C/EBPα-mediated C/EBP signaling activation in a dose-dependent manner. It was reported that C/EBP transcription factors dysregulated transcription from long terminal repeat [25]. We therefore analyzed whether HBZ could modulate HTLV-1 promoter activity through C/EBP signaling. Consistent with previous reports, overexpression of C/EBPα inhibited Tax-mediated HTLV-1 LTR activation [29]. Moreover, HBZ overcame the repression of HTLV-1 viral transcription by C/EBPα (Figure 1B). These results collectively indicate that HBZ impairs the function of C/EBPα.

### HBZ interacts with C/EBPα

**Figure 2:**
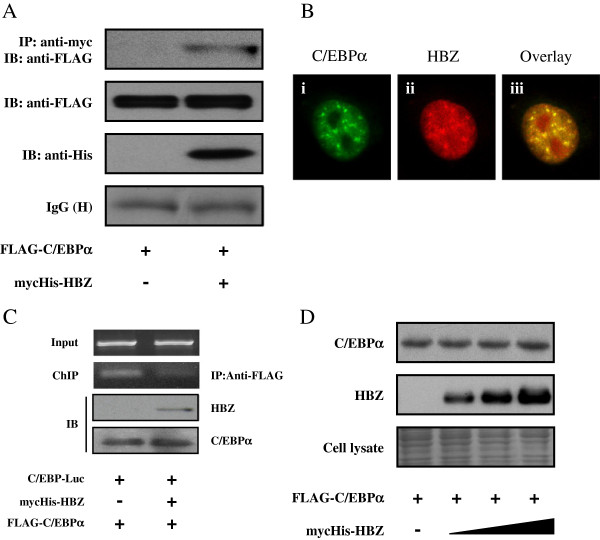
**HBZ interacted with C/EBPα protein. (A)** HBZ interacted with C/EBPα. 293T cells were cotransfected with mycHis-HBZ together with FLAG-C/EBPα. After 48 hours, cell lysates were subjected to immunoprecipitation using anti–c-Myc followed by immunoblotting using anti-FLAG. **(B)** HBZ co-localized with C/EBPα. Hela cells were transfected with mycHis-HBZ and FLAG-C/EBPα. HBZ was detected using anti–MYC Cy3 antibody (ii). C/EBPα was detected using anti–Flag-biotin and secondary Streptavidin-Alexa 488 antibody (i). The overlay of HBZ and C/EBPα is shown (iii). **(C)** HBZ decreased C/EBPα’s DNA binding capability. After transfection with mycHis-HBZ, FLAG-C/EBPα, and pC/EBPα-Luc for 48 hours, 293T cells were chromatin immunoprecipitated by anti-FLAG antibody. The precipitated DNAs and 1% of the input cell lysates were amplified by the pC/EBP-Luc specific primers. Expression of HBZ and C/EBPα was detected by Western blot (bottom panel). **(D)** HBZ could not repress the level of C/EBPα. 293T cells were transfected with expression vector of C/EBPα and various amounts of mycHis-HBZ. After 48 hours, the cell lysates were subjected to immunoblotting.

Accumulating evidences show that HBZ dysregulates signaling pathways in ATL by associating with multiple transcriptional factors [8,12-15,30,31]. To clarify the molecular mechanism by which HBZ suppresses the C/EBPα transcriptional response, we investigated whether HBZ can physically interact with C/EBPα. FLAG-tagged C/EBPα and mycHis-tagged HBZ were cotransfected into 293T cells, and an immunoprecipitation assay was performed. Figure 2A illustrates that HBZ interacted with C/EBPα. The HBZ-C/EBPα association was further analyzed by confocal microscopy. Cotransfected cells showed nuclear spots representing co-localization of HBZ and C/EBPα protein (Figure 2B). To investigate whether HBZ influences the ability of C/EBPα to bind its DNA target, we performed a ChIP assay in 293T cells that were cotransfected with C/EBP-Luc reporter together with expression vectors of HBZ and C/EBPα. The ChIP assay detected the association of C/EBPα with its responsive elements, while HBZ dramatically decreased C/EBPα’s DNA binding capability (Figure 2C). Previous reports showed that HBZ decreased the expression level of its associated proteins [12,13]. Therefore, we analyzed whether HBZ could also affect the expression of C/EBPα. As shown in Figure 2D, HBZ did not induce C/EBPα protein degradation even at high doses. In addition, C/EBPα did not influence HBZ expression (Additional file 1: Figure S1). These observations suggest that HBZ represses C/EBPα-induced transcription through physical association between HBZ and C/EBPα.

### HBZ depends on Smad3 to inhibit C/EBPα-mediated transcription

**Figure 3:**
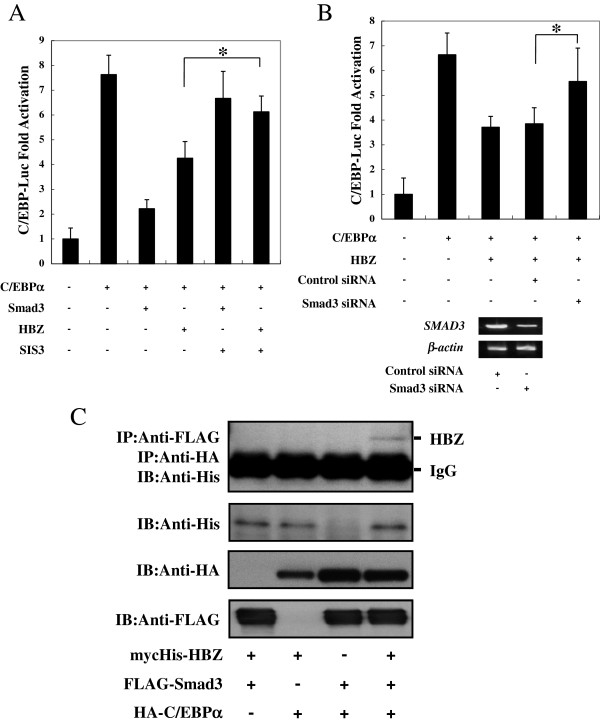
**Smad3 was involved in the suppression of C/EBPα signaling by HBZ. (A)** SIS3 overcame HBZ-induced repression of C/EBPα. Six hours after SIS3 (5 μM) treatment, Jurkat cells were cotransfected with pC/EBP-Luc (0.5 μg), phRL-TK (10 ng), pME18Sneo-HBZ (2 μg), and pCMV-Tag-C/EBPα (0.5 μg). Luciferase activity was measured 48 hours after transfection. **(B)** Reducing *SMAD3* expression by siRNA recovered HBZ mediated suppression of C/EBPα. HepG2 cells were transfected with expression vectors together with Smad3 siRNA or control siRNA. *SMAD3* mRNA expression was analyzed by RT-PCR. Luciferase activity was measured 48 hours after transfection. **(C)** HBZ, Smad3, and C/EBPα could form a ternary complex. mycHis-HBZ, FLAG-Smad3, and HA-C/EBPα were cotransfected into 293T cells. Ternary complexes were detected by sequential immunoprecipitation with anti-FLAG agarose affinity gel and anti-HA antibody, followed by immunoblotting with the His antibody.

Several reports have indicated that Smad3 interacted with C/EBP and repressed C/EBP transactivation function [32,33]. Moreover, HBZ could enhance the Smad3-mediated TGF-β pathway [14]. To determine whether Smad3 is required for HBZ to suppress C/EBPα, we analyzed the effect of SIS3, an inhibitor of Smad3, on the ability of HBZ to inhibit C/EBPα transcriptional activity. Figure 3A demonstrates that SIS3 impaired the ability of HBZ to suppress transcriptional activity through C/EBP-responsive elements. In addition, when Smad3 expression was inhibited by siRNA, the HBZ-mediated suppression of C/EBPα activity was partially restored, indicating that Smad3 functions to suppress C/EBPα signaling along with HBZ (Figure 3B). We next explored whether HBZ, C/EBPα, and Smad3 could form a ternary complex. Vectors expressing mycHis-HBZ, FLAG-Smad3, and HA-C/EBPα were cotransfected into 293T cells, and a serial immunoprecipitation assay was performed. As shown in Figure 3C, and Additional file 2: Figure S2, we detected a specific ternary complex only when the three components were coexpressed. These results together suggest that HBZ inhibits C/EBPα signaling by forming complexes of HBZ-Smad3-C/EBPα.

### Domains of HBZ responsible for suppression of C/EBPα

**Figure 4:**
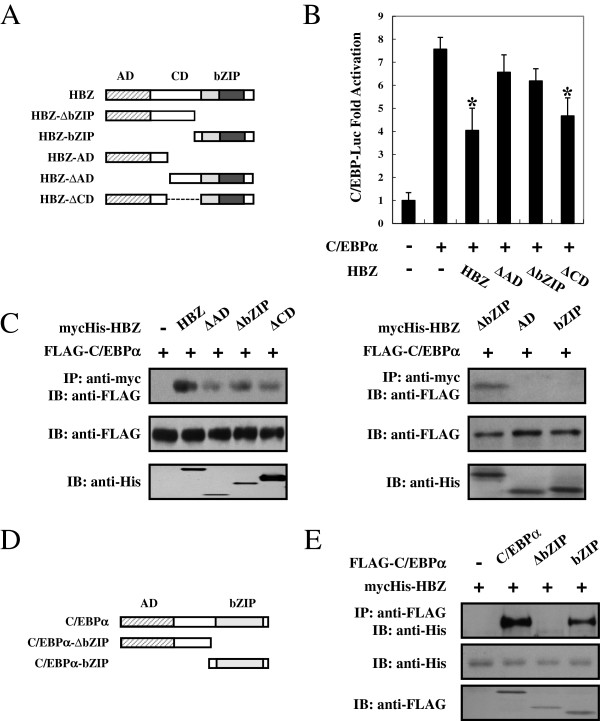
**Domains of HBZ responsible for the suppression of C/EBPα signaling. (A)** Schematic diagram of HBZ and its mutants used in this study. Characteristic domains of HBZ are indicated as follows: activation domain (AD), central domain (CD), and basic leucine zipper domain (bZIP). **(B)** Analysis of HBZ deletion mutants for their effect on C/EBPα-mediated signaling. Jurkat cells were cotransfected with pC/EBP-Luc, phRL-TK, pCMV-Tag-C/EBPα and pME18Sneo-HBZ mutants. After 48 hours, the cells were harvested and analyzed for luciferase activity. *P < 0.05; **P < 0.01. **(C)** Determination of the region of HBZ responsible for the interaction with C/EBPα. 293T cells were transfected with the indicated mycHis-HBZ mutants together with the FLAG-C/EBPα. Cell lysates were subjected to immunoprecipitation using anti–c-Myc followed by immunoblotting using anti-FLAG. **(D)** The schema of C/EBPα deletion mutants is shown. The locations of the AD domain and the bZIP domain are indicated. **(E)** Mapping the region of the C/EBPα protein necessary for interaction with HBZ. 293T cells were transfected with mycHis-HBZ and full-length or mutant FLAG-C/EBPα. At 48 hours after transfection, total cell lysates were subjected to IP using anti-FLAG followed by IB using anti–His.

Next, we evaluated the region of HBZ responsible for the inhibition of C/EBP signaling. To this end, we tested the HBZ deletion mutants shown in Figure 4A. Figure 4B demonstrated that wild-type HBZ down-regulated C/EBPα-mediated transcriptional responses. Compared with other mutants, only the HBZ ∆CD mutant exhibited suppressive activity. We mapped the region of HBZ interacting with C/EBPα in detail. As shown in Figure 4C, full-length HBZ and three of its deletion mutants (HBZ-∆AD, HBZ-∆bZIP, and HBZ-∆CD) associated with C/EBPα, while HBZ-AD and HBZ-bZIP have no binding capability. These results collectively indicate that both the AD and bZIP domains in HBZ were necessary for suppression of the C/EBPα pathway. To define which part of C/EBPα binds HBZ, we performed a co-immunoprecipitation assay with C/EBPα mutants (Figure 4D). The C/EBPα-∆bZIP mutant, which did not contain the bZIP domain, was incapable of interacting with HBZ. However, the mutant containing only the bZIP domain of C/EBPα still interacted efficiently with HBZ protein. Thus, the interaction with HBZ is mediated by the bZIP segment of C/EBPα (Figure 4E).

### C/EBPα is overexpressed in ATL

**Figure 5:**
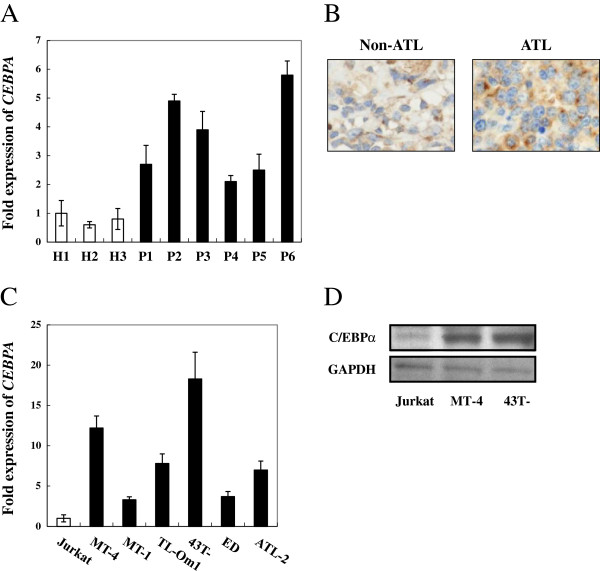
**C/EBPα was overexpressed in ATL. (A)** High expression of *CEBPA* in ATL. CD4 positive cells were isolated from PBMCs of healthy donors and ATL patients, and real-time PCR was performed to analyze the expression of *CEBPA* mRNA. H indicates healthy donors, P indicates ATL patients. **(B)** Determination of C/EBPα in ATL patient by immunohistochemical analysis. Lymph nodes of an ATL patient was fixed, and C/EBPα was subjected to immunostaining with anti-C/EBPα antibody. **(C)***CEBPA* is overexpressed in HTLV-1 associated cell lines. Quantitative analysis of *CEBPA* mRNA in HTLV-1-negative (open bars) and HTLV-1-positive cell lines (black bars) by real-time PCR. **(D)** Overepression of C/EBPα protein in HTLV-1 cell lines. Celll lystes of HTLV-1-negative and HTLV-1-positive cell lines was subjected to immunoblotting with anti-C/EBPα.

We next checked the expression level of *CEBPA* mRNA and protein in ATL. Three healthy donors and six ATL patients with different age and disease status were included in this study. CD4 positive cells were isolated from PBMCs of the clinical samples, and real-time PCR was performed to analyze the expression of *CEBPA* mRNA. Compared with normal T cells, all ATL patients constitutively expressed *CEBPA* transcript. Noticeably, the three youngest patients who suffered from acute ATL expressed higher levels of *CEBPA* compared with the other three patients (Figure 5A). Immunohistochemical analysis of lymph nodes of ATL patients showed that lymphoma cells indeed expressed C/EBPα (Figure 5B). Quantitative analyses revealed increased expression of *CEBPA* in HTLV-1-infected cell lines compared with noninfected ones (Figure 5C). Moreover, high levels of C/EBPα protein were detected in ATL cell lines (Figure 5D).

### C/EBPα expression is induced by HBZ

**Figure 6:**
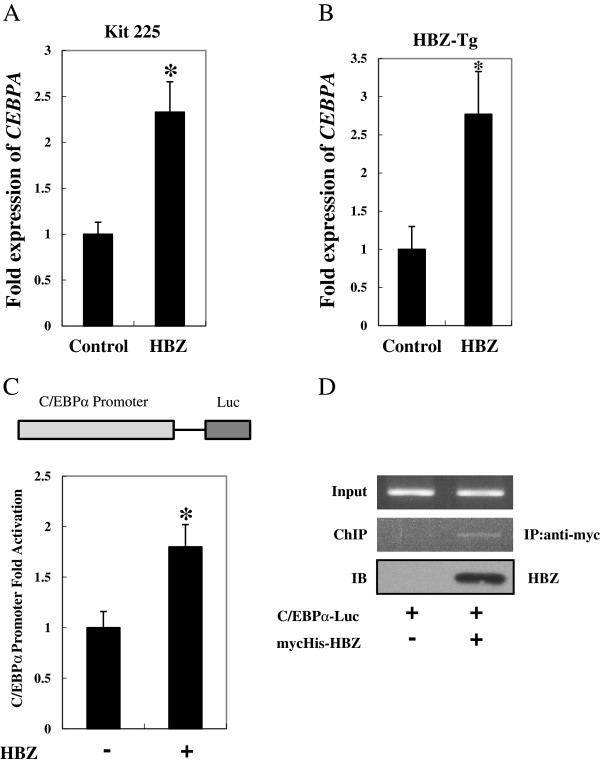
**HBZ induced C/EBPα expression.** Total RNA was extracted from control or HBZ-expressing Kit 225 **(A)** and CD4^+^ cells of HBZ transgenic mice **(B)**. Real-time PCR was performed to analyze the expression of *CEBPA* mRNA. **(C)** HBZ activated transcription of the *CEBPA* promoter. 293T cells were transfected with the C/EBPα reporter plasmid with or without the HBZ-expressing plasmid. Luciferase activity was measured 48 hours after transfection. **(D)** HBZ binds to the C/EBPα promoter. After transfection with mycHis-HBZ and C/EBPα reporter vector for 48 hours, 293T cells were chromatin immunoprecipitated by anti–c-Myc antibody. The precipitated DNAs and 1% of the input cell lysates were amplified by the specific primers for *CEBPA* promoter.

It is well established that HBZ is the only viral gene that remains intact and is constitutively expressed in all ATL cases [34]. Considering that the level of C/EBPα is elevated in ATL and HTLV-1 associated cell lines, we evaluated whether HBZ controlled the excess expression of C/EBPα. As shown in Figure 6A, the *CEBPA* gene was upregulated in Kit 225 cells, which stably express HBZ. To investigate HBZ-mediated enhancement of *CEBPA* expression *in vivo*, we studied the level of *CEBPA* in splenic CD4^+^ T cells from HBZ transgenic mice. Consistently, expression of *CEBPA* was upregulated in HBZ transgenic mice as observed *in vitro* (Figure 6B). We further analyzed the mechanism by which HBZ induced C/EBPα expression. The 2-kb fragment of the *CEBPA* promoter region was cloned into the pGL4.10 reporter vector and a luciferase assay was performed. As shown in Figure 6C, HBZ enhanced transcription from the *CEBPA* promoter. In addition, a chromatin immunoprecipitation assay detected HBZ bound to the *CEBPA* promoter (Figure 6D). These results collectively indicate that the enhanced induction of *CEBPA* expression by HBZ can be attributed, at least in part, to the association of HBZ with the *CEBPA* promoter.

### HBZ overcomes C/EBPα-mediated suppression of T-cell proliferation

**Figure 7:**
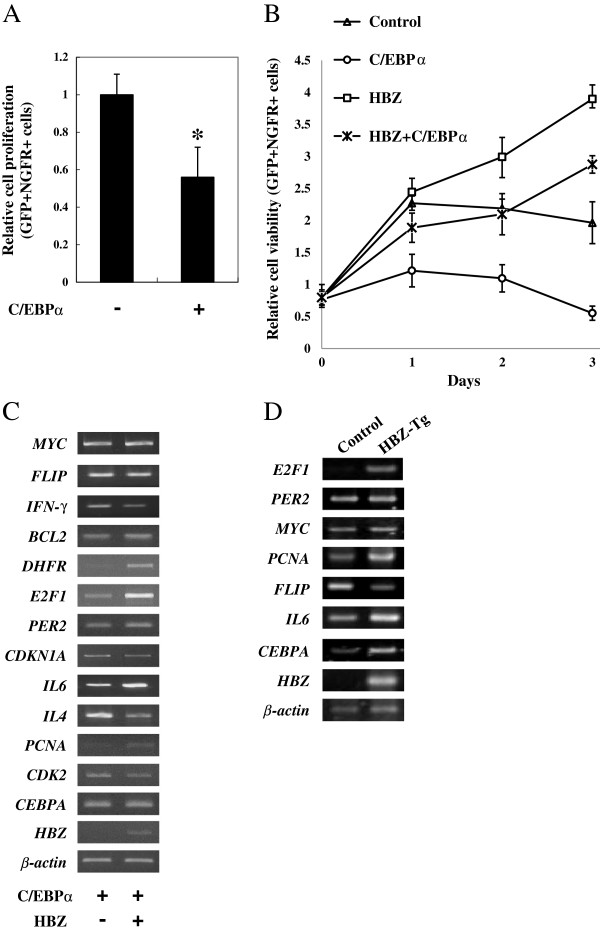
**HBZ overcame the C/EBPα-mediated growth suppression. (A)** Mouse CD4^+^CD25^-^ T cells were transduced with pGCDNsamI/GFP vector encoding C/EBPα, or with empty vector. At three days after infection, cell proliferation was analyzed by flow cytometry. **(B)** Mouse CD4^+^CD25^-^ T cells were transduced with pGCDNsamI/NGFR vector encoding HBZ together with pGCDNsamI/GFP-C/EBPα. Cells were stained with specific antibody at the time points indicated, and cell growth was detected by flow cytometry. Representative data from three independent experiments are shown. **(C)** HBZ modulated the expression of selected C/EBPα target genes. Total RNA was extracted from samples from the experiment of Figure 7B. The level of *MYC*, *FLIP*, *IFN-γ*, *BCL2*, *DHFR*, *E2F1*, *PER2*, *CDKN1A*, *IL6*, *IL4*, *PCNA*, *CDK2*, *β-actin*, *CEBPA*, and *HBZ* mRNA were analyzed by semiquantitative RT-PCR. **(D)** Transcriptional changes of selected C/EBPα target genes in CD4^+^ thymocytes from HBZ transgenic mice. After stimulating the cells with PMA plus ionomycin, the levels of *MYC*, *FLIP*, *E2F1*, *PER2*, *IL6*, *PCNA*, *β-actin*, *CEBPA*, and *HBZ* mRNA were analyzed by semiquantitative RT-PCR.

Previous studies have shown that C/EBPα inhibits cell proliferation and induces cell cycle arrest [17]. We confirmed that the growth of mouse CD4^+^ T cells was inhibited by enforced expression of C/EBPα (Figure 7A). To address whether HBZ could affect cell proliferation by suppressing C/EBPα signaling, we overexpressed HBZ and C/EBPα in primary mouse CD4^+^ T cells. Figure 7B demonstrated that C/EBPα repressed T cell proliferation, whereas HBZ-expressing cells proliferated regardless of C/EBPα. We next studied the effect of HBZ on transcription of C/EBPα-specific target genes using mouse naïve T cells expressing HBZ. Previous reports showed that C/EBPα suppressed cell proliferation by inhibiting the expression of *E2F1*, *DHFR*, and *PCNA*. When co-expressed with C/EBPα, HBZ enhanced *E2F1*, *DHFR*, *PCNA*, *FLIP*, *BCL2*, *IL6*, and suppressed *IL4* and *IFN-γ* (Figure 7C). This indicated that HBZ overcame the suppressive effect of C/EBPα on its target genes, leading to the cell growth. To investigate HBZ-mediated suppression of C/EBPα signaling *in vivo*, we studied the expression of C/EBPα-specific target genes in thymus CD4^+^ cells from HBZ transgenic mice. As shown in Figure 7D, expression of HBZ was associated with enhanced transcription of *CEBPA*, *E2F1*, *PCNA*, and *IL6* genes and suppression of *FLIP* gene; such effects were consistent with the observation in HBZ transfected naïve T cells.

There results together indicate that HBZ supports the proliferation of T cells through dysregulation of C/EBPα signaling as well as selective modulation of transcription of C/EBPα target genes.

## Discussion

After transmission, HTLV-1 increases its viral copy number by clonal proliferation of infected cells and results in the onset of ATL [5,35]. In this strategy, Tax was thought to play a critical role in increasing the number of HTLV-1-infected cells by promoting proliferation and inhibiting apoptosis [36,37]. However, because Tax is the major target of cytotoxic T lymphocytes (CTLs), it is frequently inactivated by genetic and epigenetic modifications [5,38]. Therefore, HTLV-1 has evolved mechanisms to maintain cell survival in a Tax-independent manner. We have reported that HBZ, which is consistently expressed in ATL, promotes the proliferation of T-lymphocytes *in vitro*, and increases splenic CD4^+^ T-cells in HBZ transgenic mice, indicating a role for HBZ, like tax, in the proliferation of HTLV-1 infected cells [7,31]. So far, the mechanism by which HBZ promotes proliferation of leukemic cells has not been well elucidated. Accumulating evidence shows that C/EBPα possesses the ability to arrest cell proliferation through upregulation of CDKN1A (p21) as well as direct inhibition of E2F [39]. We firstly present evidence that C/EBPα is highly expressed in ATL. However, C/EBPα’s growth-suppression function is impaired by HBZ, resulting in the proliferation of ATL cells despite C/EBPα expression. It is thus likely that HBZ may support the proliferation of HTLV-1 infected cells, whereas other mechanisms, which include dysregulation of C/EBPα signaling and selectively modulate C/EBPα target gene expression. In support of our hypothesis, we showed in this study that HBZ enhanced the expression of *E2F1*, *PCNA*, and *DHFR* genes in C/EBPα-expressing cells and did not interfere with *MYC*, *CDKN1A*, and *CDK2* expression, contrary to the effect of C/EBPα alone [7].

Apart from the growth suppression function, C/EBP family proteins have oncogenic properties [17,21]. Consistent with our findings, recent studies reported that overexpression of C/EBPα occurs in cancer, such as B precursor acute lymphoblastic leukemia (ALL) and a subset of human hepatocellular carcinomas (HCCs) [22,40]. Importantly, C/EBPα induces *BCL2* and *FLIP* gene expression in cooperation with NF-κB p50, allowing cancer cells to escape apoptosis [23]. We showed here that C/EBPα was overexpressed in ATL, whereas its growth-suppressive function was impaired by the effect of HBZ. In this regard, it is meaningful to raise the question: why do ATL cells need high levels of C/EBPα? It has been reported that HBZ suppressed apoptosis of HTLV-1 infected cells, while the underlying mechanism is still unknown. As shown in Figure 7C, HBZ selectively suppressed the level of C/EBPα target genes which related with cell growth, but did not inhibit the C/EBPα-induced expression of anti-apoptotic genes including *BCL2* and *FLIP*, suggesting that HBZ may fulfill its anti-apoptotic function through dysregulation of C/EBPα signaling.

Immunodeficiency in ATL patients is pronounced, and results in frequent opportunistic infections by various pathogens [41,42]. As a mechanism of this immunodeficiency, HBZ has been shown to inhibit CD4 T-cell responses, resulting in impaired host immunity *in vivo*[31,43]. Further study demonstrated that HBZ transgenic mice, which expressed excess amount of C/EBPα, were vulnerable to opportunistic pathogens [31]. It was reported that a population of PD-1+ memory phenotype CD4^+^ T cell underlies the global depression of the T cell immune response, and such features are attributable to an unusual expression of C/EBPα [44]. Like C/EBPα, C/EBPβ acts as a master regulator of the tolerogenic and immunosuppressive environment induced by cancer [45]. Thus, our results now open the possibility that HBZ may induce the expression of C/EBPα, leading to immunodeficiency in ATL, and perhaps to oncogenesis. Further studies on C/EBP signaling in ATL are necessary to clarify its roles.

Many viruses have developed distinct strategies to modulate C/EBPα signaling using their own viral proteins. Examples include hepatitis B virus pX; Epstein-Barr virus BZLF; as well as human immunodeficiency virus TAT and Vpr [46-48]. Like HBZ, the HBV pX and EBV BZLF protein prevent C/EBP-mediated activation by interacting directly with C/EBP family members. Similar upregulation of C/EBP expression has been reported for other viruses, including hepatitis C virus, Kaposi’s sarcoma-associated herpes virus, and human immunodeficiency virus [49-51]. These findings show that dysregulation of C/EBP pathways are common among different viruses, suggesting that these activities are critical for viral persistence and oncogenesis.

Accumulating evidences show that HBZ’s oncogenic function can be attributed, at least in part, to its selective regulation of multiple signaling pathways in ATL [13-15,30,31]. For example, HBZ inactivates classical NF-κB signaling without inhibiting the alternative pathway, helping cells to evade senescence and supporting cell proliferation [13,52]. Similarly, the negative effects of transcription factors which include ATF3, Wnt5a, and Smad3, were impeded by HBZ, leaving these factors to elude host immune attack and promote cell proliferation [14,15,30]. In this study, we found that HBZ selectively impaired the growth suppression function of C/EBPα, rendering the immunosuppressive and anti-apoptotic effect of C/EBPα predominant. HTLV-1 might escape from host immune surveillance and induce cell proliferation by thus selectively modulating signaling pathways, promoting viral reproduction, and also ATL.

It has been reported that HBZ is not able to form stable homodimers and is therefore dependent on heterodimerization with other proteins to control gene transcription [53]. Thus, the function of HBZ depends, at least in part, on its binding partner. Indeed, HBZ selectively suppressed the classical NF-κB pathway through inhibiting DNA binding of p65 as well as PDLIM2-dependent p65 degradation. The specificity of PDLM2 E3 ligase in targeting p65 protein, but not p52 of the alternative pathway, may possibly explain why HBZ selectively inhibits the classical pathway of NF-κB [13]. Similarly, we showed in this study that HBZ inhibited C/EBPα signaling via recruitment of Smad3. Because the association with Smad proteins is crucial for C/EBPα in determining its target genes as well as transcriptional outcome, it is likely that the function of HBZ-Smad3-C/EBPα complexes depends on the capacity of HBZ to recruit Smad3-C/EBPα heterodimers onto the DNA target [32,54].

## Conclusion

We showed that HBZ impaired the growth suppression function of C/EBP signaling by physically interacting with C/EBPα. HTLV-1 may take advantage of this mechanism to allow the infected cells to proliferate *in vivo*.

## Methods

### Cell culture, mice, and clinical samples

293T, Hela, and HepG2 cells were grown in Dulbecco’s modified Eagle’s medium (DMEM) supplemented with 10% fetal bovine serum (FBS) and antibiotics. HTLV-1 immortalized cell lines (MT-4), ATL cell lines (MT-1, ATL-2, ATL-43T, ED, and TL-Om1), and T-cell lines not infected with HTLV-1 (Jurkat) were cultured in RPMI 1640 supplemented with 10% FBS and antibiotics. Kit 225 cells stably expressing HBZ were maintained as described previously [7]. C57BL/6J mice were purchased from CLEA Japan (Tokyo, Japan). Transgenic HBZ mice expressing HBZ specifically in CD4^+^ cells have been described [55]. Peripheral blood mononuclear cells (PBMCs) were isolated from ATL patients (n = 6), and healthy volunteers (n = 3). Details of clinical samples are shown in Additional file 3: Table S1.The study of clinical samples was conducted according to the principles expressed in the Declaration of Helsinki and approved by the Institutional Review Board of Kyoto University (844 and E-921). All patients provided written informed consent for the collection of samples and subsequent analysis.

### Plasmids

The pC/EBP-Luc construct contains three tandem C/EBP binding sites and was purchased from Stratagene (Heidelberg, Germany). phRL-TK was purchased from Promega (Madison, WI). Reporter vector pLTR-Luc as well as expression plasmids for Tax, Smad3, HBZ, and HBZ deletion mutants were prepared as previously described [7,13,14]. Expression vectors for C/EBPα and its deletion mutants were generated by PCR.

### Luciferase assay

Jurkat cells were plated on 6-well plates at 3.5×10^5^ cells per well. After 24 hours, cells were transfected with the indicated luciferase plasmid DNA. Forty-eight hours after transfection, a luciferase reporter assay was performed as previously described [13]. For the C/EBPα reporter assay, the *CEBPA* gene promoter was cloned into the pGL4.1 vector. Luciferase values were normalized to renilla luciferase and expressed as the mean of a triplicate set of experiments ± SD.

### Immunoprecipitation and immunoblotting

293T cells were transfected with the indicated combinations of expression vectors by *Trans*IT-LT1 (Mirus, Madison, WI). Tagged proteins were immunoprecipitated by anti–c-Myc (clone 9E10, Sigma-Aldrich, St Louis, MO), anti-HA (12CA5, Roche, Mannheim, Germany) or anti-FLAG M2 (Sigma-Aldrich) antibodies, and analyzed by Western blot. Serial immunoprecipitation was performed as described previously [14]. Other antibodies used were as follows: anti-mouse immunoglobulin G (IgG), and anti-rabbit IgG were from GE Healthcare Life Sciences, and anti-C/EBPα from Santa Cruz Biotechnology (Santa Cruz, CA).

### Immunofluorescence analysis

Hela cells were transfected with expression vectors using *Trans*IT-LT1. Forty-eight hours after transfection, HBZ protein was detected using anti–c-MYC Cy3 (clone 9E10, Sigma-Aldrich). C/EBPα was detected using anti–FLAG-biotin (Sigma-Aldrich) and secondary Streptavidin-Alexa 488 antibody (Invitrogen, Carlsbad, CA). Fluorescence was observed with a confocal microscope system (Leica, Wetzlar, Germany) as described previously [14].

### Chromatin immunoprecipitation assay

293T cells were transfected with the HBZ and C/EBPα expression vectors together with pC/EBP-Luc reporter vector. Forty-eight hours after transfection, chromatin immunoprecipitation (ChIP) assay was performed as previously described [14]. Precipitated DNA was amplified by PCR using primers specific for the pC/EBP-Luc plasmid. Sequences for the primer set were 5′-TCACTGCATTCTAGTTGTGG-3′ and 5′-CCATCCTCTAGAGGATAGA-3′.

### Semiquantitative RT-PCR and quantitative real-time PCR

Total RNA was isolated using Trizol Reagent (Invitrogen) according to the manufacturer’s instructions. We reverse transcribed total RNA into single-stranded cDNA with SuperScript III reverse transcriptase (Invitrogen). For semiquantitative PCR, cDNA was amplified by increasing PCR cycles using forward (F) and reverse (R) primers specific to the target genes. In the real-time PCR experiment, cDNA product was quantified with Power SYBR Green PCR Master Mix and StepOnePlus Real Time PCR System (Life technologies). Endogenous *β-actin* mRNA was quantified to normalize the amount of cDNA load. The specific primers used can be found in Additional file 4: Table S2.

### Immunohistochemical analyses

The tissue specimens were obtained from human lymph nodes filed at the Department of Pathology at Kurume University. Tissue samples were fixed in 10% formalin in phosphate buffer and then embedded in paraffin and analyzed by immunohistochemical methods to determine C/EBPα expression. Images were captured using a Provis AX80 microscope equipped with an OLYMPUS DP70 digital camera, and detected using a DP manager system (Olympus, Tokyo, Japan). The study of clinical samples was approved by the local research ethics committee of Kurume University.

### Small interfering RNA (siRNA) transfection

siRNA targeted to human Smad3 was synthesized according to a previous report [56]. HepG2 cells were transfected with expression vectors and siRNA using *Trans*IT-LT1 according to the manufacturer’s instructions. RT-PCR detected *SMAD3* 48 hours after transfection.

### Retroviral constructs and transduction

pGCDNsamI/NGFR-HBZ and pGCDNsamI/GFP-C/EBPα retroviral constructs were generated by cloning HBZ and C/EBPα cDNA into the pGCDNsamI/NGFR and pGCDNsamI/GFP vectors respectively. Transfection of Plat-E packaging cell line was performed as described [57]. Mouse splenocytes were enriched for CD25^-^CD4^+^ cells with a CD4 T lymphocyte enrichment set (BD Biosciences) with the addition of biotinylated anti-CD25 antibody (BD Biosciences), and activated by APCs in the presence of anti-CD3 antibody and human rIL-2 in 12-well plates. After 24 hours, activated T cells were transduced with viral supernatant and polybrene, and centrifuged at 3,000 rpm for 60 minutes. Cells were subsequently cultured in medium supplemented with rIL-2.

### Flow cytometric analysis

Murine cells were washed with PBS containing 1% FBS. After centrifugation, cells were treated with APC-conjugated anti-human NGFR antibody (BD Biosciences) for 30 minutes. After being washed with PBS, the cells were analyzed with a flow cytometer (BD FACSCanto II, BD Biosciences).

### Statistical analyses

Statistical analyses were performed using the unpaired Student *t* test.

## Competing interests

The authors declare that they have no competing interests.

## Authors’ contributions

This study was designed by TZ, AC, and MM. Laboratory analysis was performed by TZ, AC, LX, and JY. Data analysis was performed by TZ, AC, LX, JY, and MM. Clinical samples and data were provided by KO. TZ, AC, and MM wrote the paper. All authors read and approved the final manuscript.

## Supplementary Material

Additional file 1: Figure S1 C/EBPα did not influence HBZ expression. 293T cells were transfected with expression vector of HBZ and increasing amounts of C/EBPα. After 48 hours, the cell lysates were subjected to Western blot. Click here for file

Additional file 2: Figure S2 HBZ, Smad3, and C/EBPα formed a ternary complex. mycHis-HBZ, FLAG-Smad3, and HA-C/EBPα were cotransfected into 293T cells. After 48 hours, cell lysates were subjected to immunoprecipitation using anti–c-Myc or anti-FLAG followed by immunoblotting using anti-FLAG, anti-His, and anti-HA antibody. Click here for file

Additional file 3: Table S1 List of healthy donors and ATL patients. The information of six ATL patients and three healthy volunteers are listed. Click here for file

Additional file 4: Table S2 List of primers for semi-quantitative RT-PCR and quantitative real-time PCR. We performed semi-quantitative RT-PCR and quantitative real-time PCR using the following primers. Click here for file
