# Supplementary figures and images for: HTLV-1 bZIP factor supports proliferation of adult T cell leukemia cells through suppression of C/EBPα signaling
Source: Retrovirology. 2013 Dec 21;10:159. doi: 10.1186/1742-4690-10-159 (PMC3880043; doi:10.1186/1742-4690-10-159)

## Slide 1
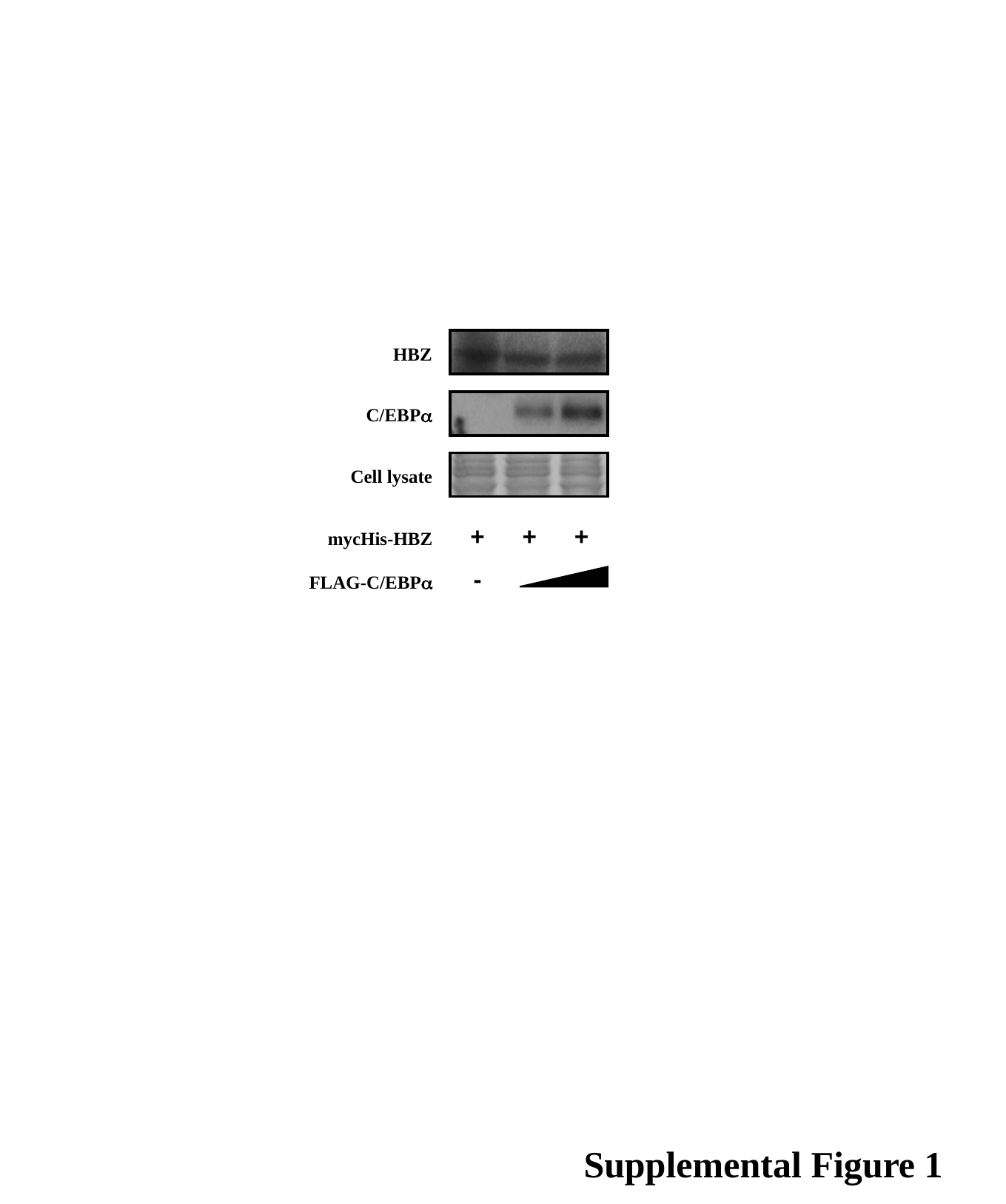

HBZ
C/EBPa
Cell lysate
| + | + | + |
| --- | --- | --- |
| - | | |
mycHis-HBZ
FLAG-C/EBPa
Supplemental Figure 1

Supplement: Additional file 1: Figure S1 — C/EBPα did not influence HBZ expression. 293T cells were transfected with expression vector of HBZ and increasing amounts of C/EBPα. After 48 hours, the cell lysates were subjected to Western blot. [file 1742-4690-10-159-S1.pptx]
