# Supplementary material for: HTLV-1 bZIP factor supports proliferation of adult T cell leukemia cells through suppression of C/EBPα signaling
Source: Retrovirology. 2013 Dec 21;10:159. doi: 10.1186/1742-4690-10-159 (PMC3880043; doi:10.1186/1742-4690-10-159)
Supplement: Additional file 2: Figure S2 — HBZ, Smad3, and C/EBPα formed a ternary complex. mycHis-HBZ, FLAG-Smad3, and HA-C/EBPα were cotransfected into 293T cells. After 48 hours, cell lysates were subjected to immunoprecipitation using anti–c-Myc or anti-FLAG followed by immunoblotting using anti-FLAG, anti-His, and anti-HA antibody. [file 1742-4690-10-159-S2.pptx]

## Slide 1
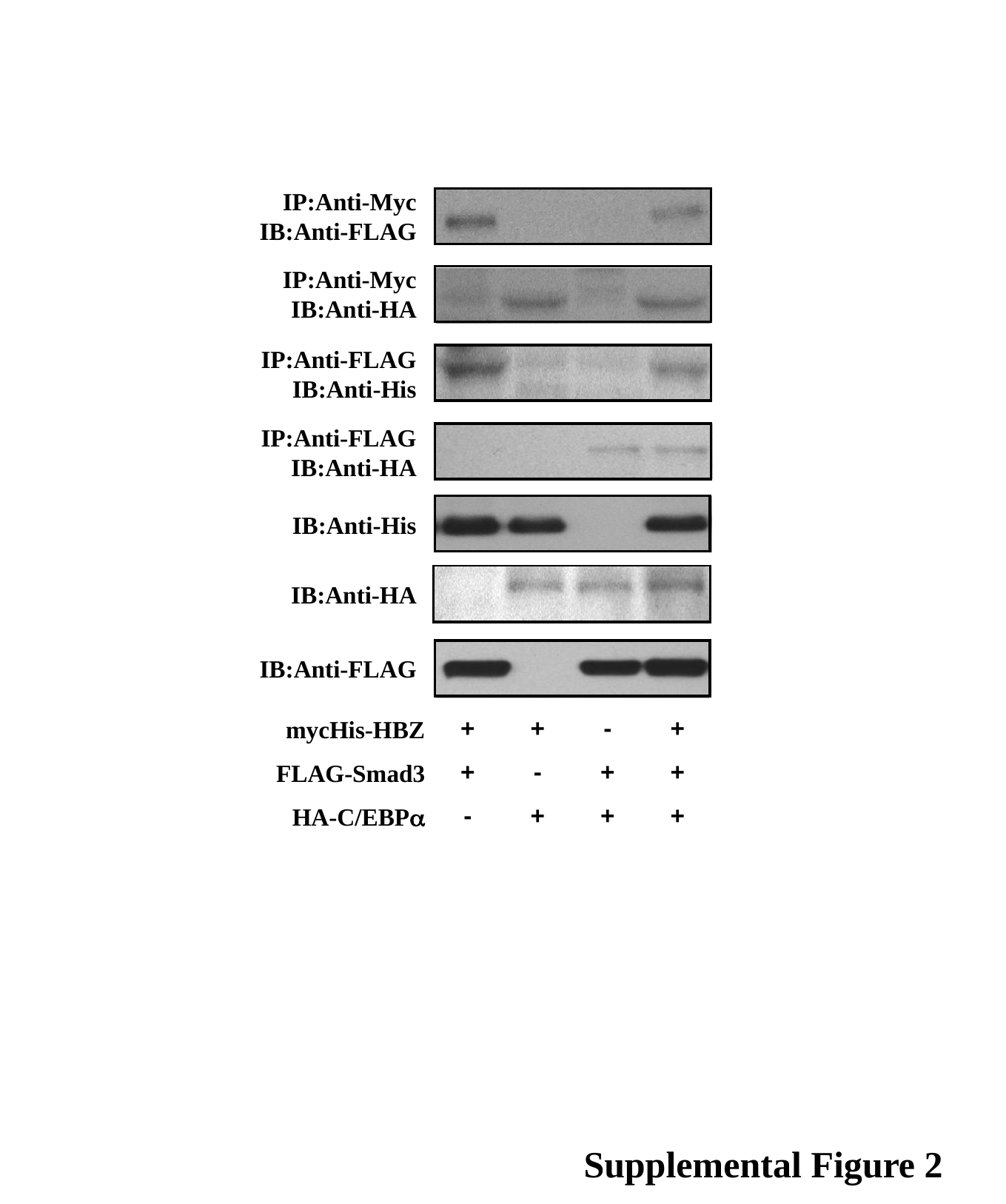

IP:Anti-Myc
IB:Anti-FLAG
IP:Anti-Myc
IB:Anti-HA
IP:Anti-FLAG
IB:Anti-His
IP:Anti-FLAG
IB:Anti-HA
IB:Anti-His
IB:Anti-HA
IB:Anti-FLAG
| + | + | - | + |
| --- | --- | --- | --- |
| + | - | + | + |
| - | + | + | + |
mycHis-HBZ
FLAG-Smad3
HA-C/EBPa
Supplemental Figure 2
