# Supplementary material for: HTLV-1 bZIP factor supports proliferation of adult T cell leukemia cells through suppression of C/EBPα signaling
Source: Retrovirology. 2013 Dec 21;10:159. doi: 10.1186/1742-4690-10-159 (PMC3880043; doi:10.1186/1742-4690-10-159)
Supplement: Additional file 3: Table S1 — List of healthy donors and ATL patients. The information of six ATL patients and three healthy volunteers are listed. [file 1742-4690-10-159-S3.pptx]

## Slide 1
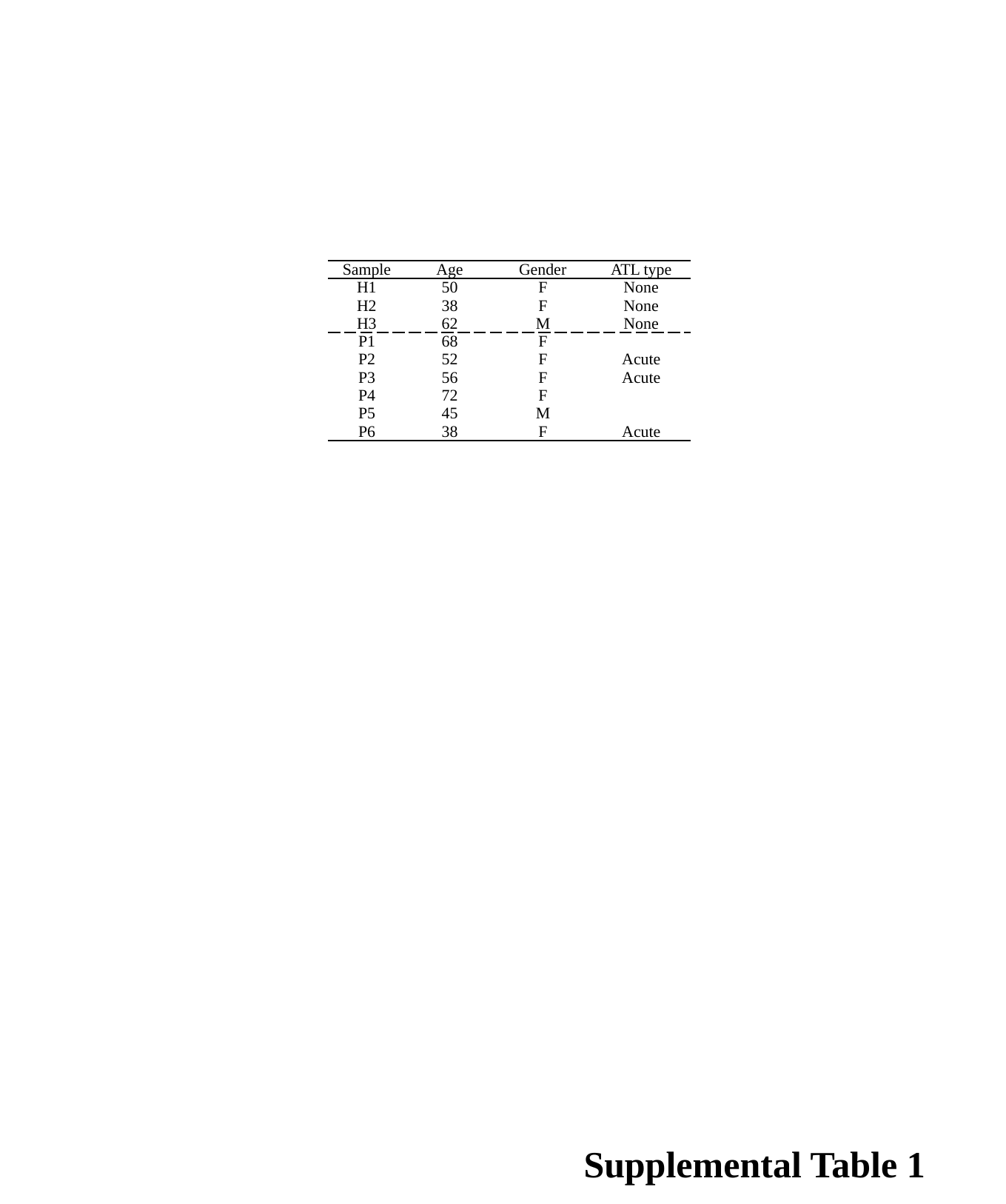

| Sample | Age | Gender | ATL type |
| --- | --- | --- | --- |
| H1 | 50 | F | None |
| H2 | 38 | F | None |
| H3 | 62 | M | None |
| P1 | 68 | F | |
| P2 | 52 | F | Acute |
| P3 | 56 | F | Acute |
| P4 | 72 | F | |
| P5 | 45 | M | |
| P6 | 38 | F | Acute |
Supplemental Table 1
