# Supplementary material for: HTLV-1 bZIP factor supports proliferation of adult T cell leukemia cells through suppression of C/EBPα signaling
Source: Retrovirology. 2013 Dec 21;10:159. doi: 10.1186/1742-4690-10-159 (PMC3880043; doi:10.1186/1742-4690-10-159)
Supplement: Additional file 4: Table S2 — List of primers for semi-quantitative RT-PCR and quantitative real-time PCR. We performed semi-quantitative RT-PCR and quantitative real-time PCR using the following primers. [file 1742-4690-10-159-S4.pptx]

## Slide 1
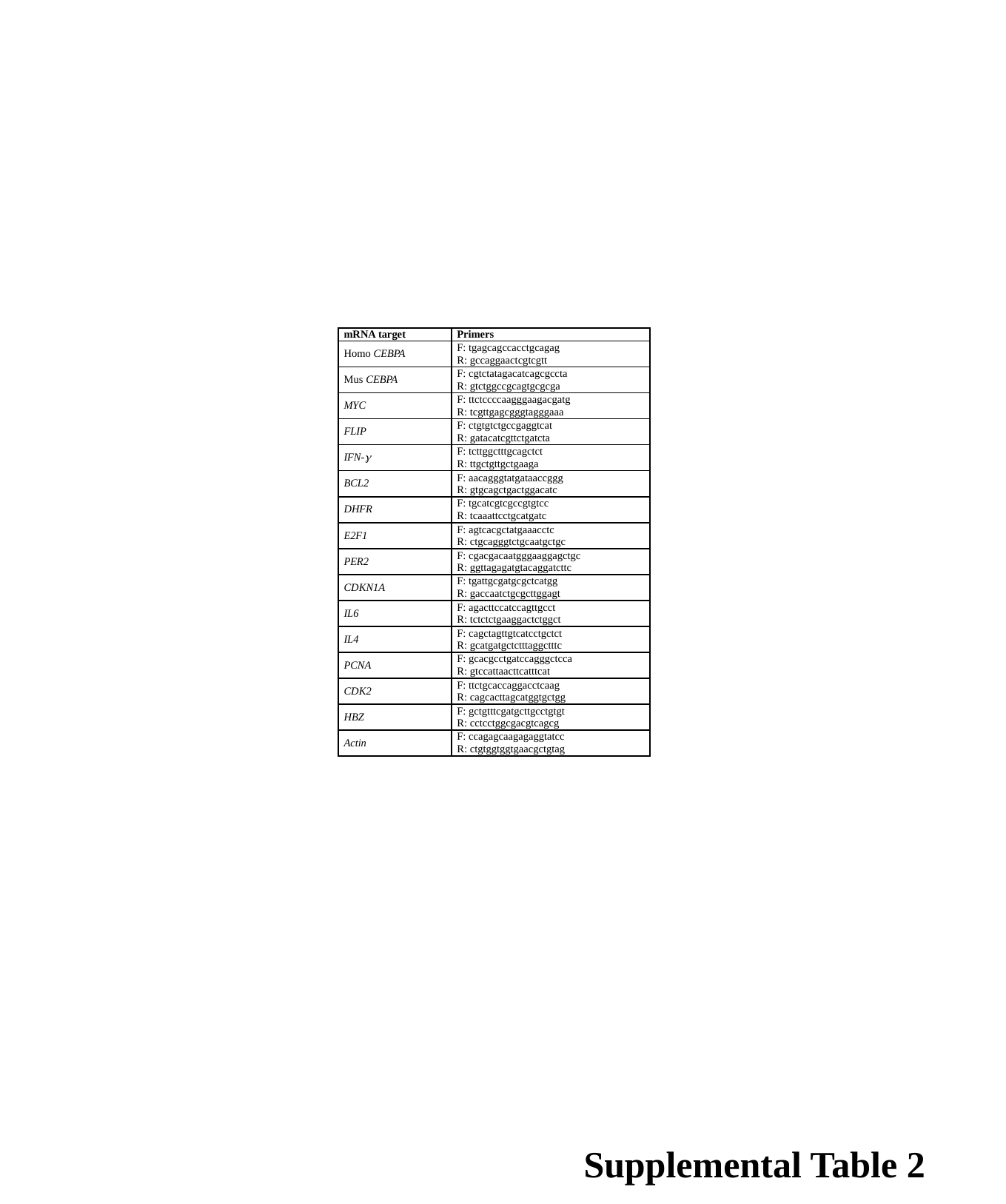

| mRNA target | Primers |
| --- | --- |
| Homo CEBPA | F: tgagcagccacctgcagag R: gccaggaactcgtcgtt |
| Mus CEBPA | F: cgtctatagacatcagcgccta R: gtctggccgcagtgcgcga |
| MYC | F: ttctccccaagggaagacgatg R: tcgttgagcgggtagggaaa |
| FLIP | F: ctgtgtctgccgaggtcat R: gatacatcgttctgatcta |
| IFN-g | F: tcttggctttgcagctct R: ttgctgttgctgaaga |
| BCL2 | F: aacagggtatgataaccggg R: gtgcagctgactggacatc |
| DHFR | F: tgcatcgtcgccgtgtcc R: tcaaattcctgcatgatc |
| E2F1 | F: agtcacgctatgaaacctc R: ctgcagggtctgcaatgctgc |
| PER2 | F: cgacgacaatgggaaggagctgc R: ggttagagatgtacaggatcttc |
| CDKN1A | F: tgattgcgatgcgctcatgg R: gaccaatctgcgcttggagt |
| IL6 | F: agacttccatccagttgcct R: tctctctgaaggactctggct |
| IL4 | F: cagctagttgtcatcctgctct R: gcatgatgctctttaggctttc |
| PCNA | F: gcacgcctgatccagggctcca R: gtccattaacttcatttcat |
| CDK2 | F: ttctgcaccaggacctcaag R: cagcacttagcatggtgctgg |
| HBZ | F: gctgtttcgatgcttgcctgtgt R: cctcctggcgacgtcagcg |
| Actin | F: ccagagcaagagaggtatcc R: ctgtggtggtgaacgctgtag |
Supplemental Table 2
